# Morphological characterization of a human glioma cell l ine

**DOI:** 10.1186/1475-2867-5-13

**Published:** 2005-05-10

**Authors:** Camila ML Machado, André Schenka, José Vassallo, Wirla MSC Tamashiro, Estela M Gonçalves, Selma C Genari, Liana Verinaud

**Affiliations:** 1 Department of Microbiology and Immunology, Biology Institute, State University of Campinas, Campinas, São Paulo, Brazil; 2 Department of Pathology, School of Medicine, State University of Campinas, Campinas, São Paulo, Brazil; 3 Department of Cellular Biology, Biology Institute, State University of Campinas, Campinas, São Paulo, Brazil

## Abstract

A human malignant continuous cell line, named NG97, was recently established in our laboratory. This cell line has been serially subcultured over 100 times in standard culture media presenting no sign of cell senescence. The NG97 cell line has a doubling time of about 24 h. Immunocytochemical analysis of glial markers demonstrated that cells are positive for glial fibrillary acidic protein (GFAP) and S-100 protein, and negative for vimentin. Under phase-contrast microscope, cultures of NG97 showed cells with variable morphological features, such as small rounded cells, fusiform cells (fibroblastic-like cells), and dendritic-like cells. However, at confluence just small rounded and fusiform cells can be observed. At scanning electron microscopy (SEM) small rounded cells showed heterogeneous microextentions, including blebs and filopodia. Dendritic-like cells were flat and presented extensive prolongations, making several contacts with small rounded cells, while fusiform cells presented their surfaces dominated by microvilli.

We believe that the knowledge about NG97 cell line may be useful for a deeper understanding of biological and immunological characteristics of gliomas.

## Background

Malignant gliomas are the most common type of brain tumor in adults. These tumors are highly invasive and despite multi-modality treatment the mean survival of patients is still less than 1 year.

Cultures of malignant cells represent an excellent and permanent material for studying the biology of these tumors as, for example, specific antigens characterization, bioactive factors produced, determination of cellular proliferation, and heterogeneity of genotypic and phenotypic characteristics (Pohl et al. 1999; Tsujino et al. 1997; Bodmer et al. 1989; Di Tomaso et al. 2000; Halfter et al. 1998; Bigner et al. 1981).

Recently, we have established a human glioma cell line from tissue obtained from a patient diagnosed with glioblastoma multiforme of the right temporal lobe. Histological examination revealed a grade III astrocytoma according to the WHO classification. This cell line, called NG97, has been sub-cultured in standard culture media without feeder layer or collagen coatings. The injection of NG97 cells into congenitally athymic mice induce the formation of solid tumor masses that can be retransplanted every 4 weeks. These tumors present features of malignant gliomas characterized by cell pleomorphism, necrosis and aggressive growth (Grippo et al. 2001).

The present work was undertaken to study growth kinetics, expression of marker proteins and morphological characteristics of early passaged cells present in the NG97 cell line.

## Results

### Markers

**Figure 1:**
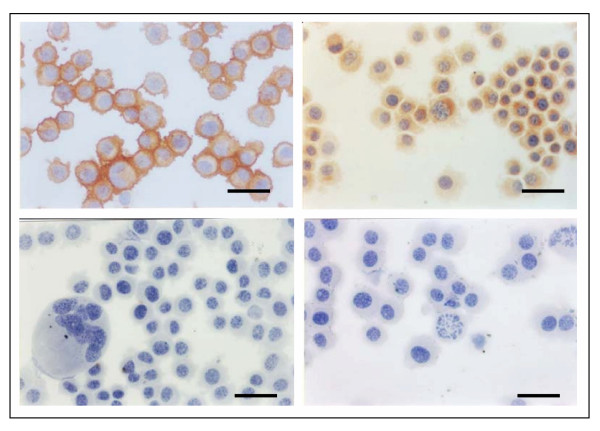
Immunocytochemical staining in NG97 cell line at passage 15. Note the immunopositive staining for GFAP (A) and S100 protein (B). On the other hand, cells were vimentin-negative stained (C). A negative control is also showed (D). Scale bar = 25 μm

Immunocytochemical analysis of glial markers in the NG97 cells demonstrated that a large number of cells were positive for GFAP and S100 protein (Figure 1A and 1B, respectively). GFAP presents a diffuse perinuclear condensation, and S-100 protein is uniformly observed in the cytoplasm and irregularly observed in the nucleus of some cells. On the other hand, vimentin was undetectable in this cell line (Figure 1C). Figure 1D shows a representative control of all immunocytochemical experiments.

### Microscopy Studies

**Figure 2:**
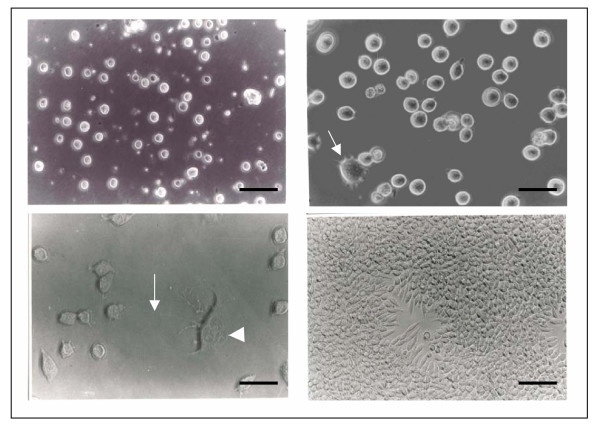
Phase contrast micrographs of NG97 cells. (A) small rounded cells growing as floating aggregates; (B) dendritic-like cells appear in the culture (→); (C) a dendritic-like cell with an extensive cytoplasmatic prolongation (→) and extra numerary nucleous (▶); (D) confluent monolayer with small rounded and fibroblastic-like cells. Scale bar = 50 μm (A and D); 25 μm (B and C).

Initially, NG97 cells formed mainly floating aggregates in the culture flasks and only small, rounded cells were seen (Figure 2A). At the 13^th ^passage dendritic-like cells appear in the culture (Figure 2B). These cells present extensive prolongations making several contacts with small rounded cells and showed extra numerary nucleous (Figure 2C). As the cultures became dense, a third cellular type appears presenting a fusiform morphology (fibroblastic-like cells). At confluence, just small and fusiform cells can be observed in the culture (Figure 2D).

**Figure 3:**
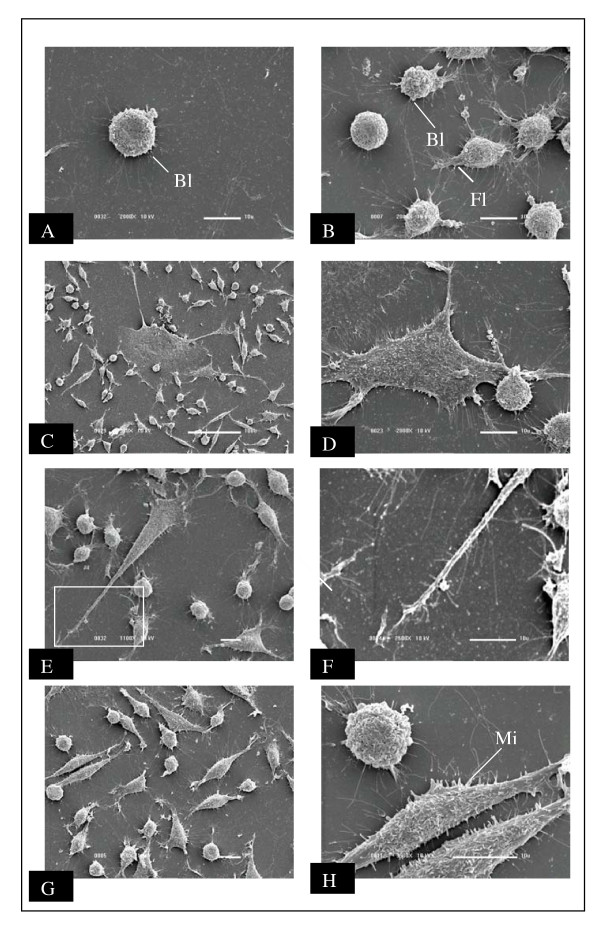
Scanning electron micrograph of NG97 cell line. A, B: small rounded cells presenting blebs (Bl) and filopodia (Fi) on their surfaces; C, D, E: dendritic-like cells with extensive cytoplasmatic prolongations. The area in the rectangle is shown at higher magnification in F; G: culture with two morphologic distinct cellular types; H: fibroblastic-like cells presenting microvilli (Mi) on the membrane surface.

Scanning electron microscopy of small rounded cells showed heterogeneity of cytoplasmatic prolongation, including blebs and filopodia (Figure 3A and 3B). Dendritic-like cells are illustrated in Figures 3C through 3F. These cells presented high degree of cellular flattening, absence of blebs and, numerous and extensive cytoplasmatic prolongations. They were attached to the substrate making contact with small rounded cells. The third morphologically distinct cell type is presented in Figures 3G and 3H. These fusiform cells presented numerous microvilli on surfaces.

### Growth kinetics

**Figure 4:**
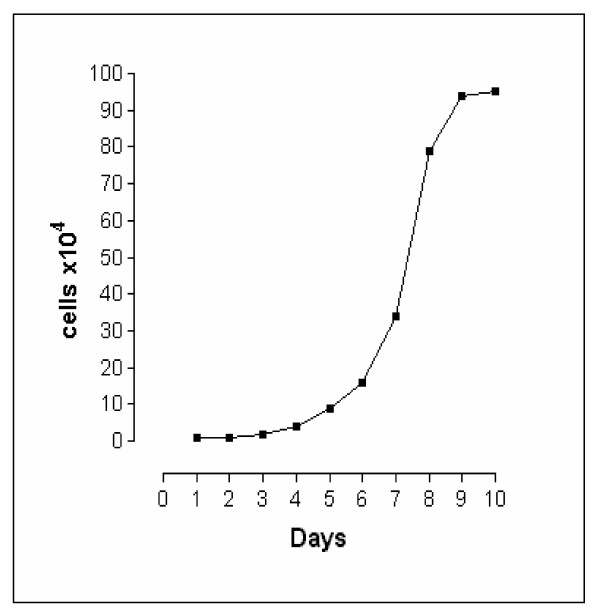
Growth curve of NG97 cell line.

Until the 13^th ^passage, when just small rounded cells were seen in the culture, a slow growth rate was observed (data not shown). At 13^th ^passage, when the two other cell types appeared in the culture, the cells entered into an exponential growth phase. The population doubling time of NG97 cell line was about 24 h at 37°C and the saturation cell density was reached at 10 × 10^5 ^cells/cm^2 ^(Figure 4). The high growth rate was observed for the successive passages

## Discussion

In this study basic characteristics of NG97 cell line are described. The investigated cell line was within passage 13 to 15. Our results show that NG97 cell line retains the expression of GFAP, which is a reliable marker of astrocytic cells, and S100 protein that was originally identified as brain specific (Moore, 1965). Literature has shown, by and large, a negative correlation between the degree of malignancy and expression of GFAP and S100 protein in the majority of human gliomas (Jacques et al., 1981; Duffy et al., 1982). However, NG97 cells are tumorigenic in nude mice, indicating that cells are neoplastic and malignant. Besides, these two biochemical markers are present in the xenografts of NG97 cells in nude mice (Grippo et al., 2001). Interestingly, the vimentin that has been identified in some human glioma cell lines (Roessmann et al. 1983; Rutka et al. 1998) was not detectable in NG97 cells. Hedberg and Chen (1986) found that a human adrenal tumor cell line, named SW-13, expressed vimentin filaments and clones derived from these cells were characterized as lacking any detectable cytoplasmic intermediate filaments (vim^-^). Later, Sarria et al. (1994) demonstrated that the nuclei of the SW-13 vim^- ^cells often appeared to be highly folded, forming prominent lobes and clefts. However, the authors also showed that the effect of vimentin filaments on the invaginations or folding in the nucleus is not an absolute, and raise the possibility that this nuclear configuration could be an indirect effect of a metabolic difference between cells that contain or lack organized vimentin filaments. To all appearances, our results indicate that in NG97 cell line the absence of an organized vimentin filament network does not affect the shape of the nucleus.

Heterogeneous cell types can be found in NG97 cultures. At early passages, cultures grew slower and presented only the small, rounded cells. At 13^th ^passage, dendritic-like cells appear in the culture. It is not clearly for us the exact events that lead to the appearance of this cell type in the culture. We hypothesize that one small, rounded cell accumulates unbalanced divisions forming an extra numerary nucleous cell that secretes some products capable to induce alterations on the other cells. More elaborate experiments would test this possibility. In addition, dendritic-like cells present numerous and extensive cytoplasmatic prolongations, which may be associated with communication between this cell and the small ones. It seems also that dendritic-like cells provide an anchorage to the small rounded cells, which in turn present an increase in the filopodias to ameliorate the substrate connections. Of note, when dendritic-like cells appear in the culture we have noted an increase of the cellular growth rate. Future analyses should test if dendritic-like cells are able to modulate cell growth.

Fusiform cells appear when the culture becomes dense. These cells are majority in confluent monolayer cultures and present a large number of microvilli on the surface that propitiates an intimate contact with the environment. In the same way, further studies of this cell will help to unveil more NG97 cell line secrets.

## Conclusion

NG97 cells grow *in vitro *as three sub populations with distinct morphological appearance and, undoubtedly, constitute a glial-committed cell line since are positive for GFAP and S-100 protein. Until 13^th ^passage only small rounded cells were seen in culture and the growth kinetics was very slow. From this point, two other cell types presenting dendritic and fibroblastic characteristics could be observed and results were evident for overgrowth of cells. The possibility that these cells are able to modulate cell growth can not be discarded and are now under investigation in our laboratory.

This cell line may prove useful for cellular and molecular studies as well as in studies of gliomas treatment.

## Methods

### Glioma Culture

NG97 cells were grown in plastic flasks (25 cm^2^) with RPMI 1640 medium (Sigma Chemical Co., St Louis, MO), supplemented with 50 μM 2-ME, 2 mM L- glutamine, 100 μg/mL garamycin and 20% inactivated fetal bovine serum (complete medium). The cultures were incubated at 37°C in an atmosphere containing 95% air and 5% CO_2_. The medium was changed after intervals of 48 hs and when the culture reached confluence, the subculture was performed by treatment with 0.05% trypsin and 0.02% ethylenediaminetetraacetic acid (EDTA).

### Immunocytochemistry

Immunocytochemical analysis of glial markers (GFAP, vimentin and S-100 protein) was performed by using specific antibodies purchased from Dako Envision+ Systems/HRP (Dako Corporation, Carpinteria, CA). Briefly, cultured NG97 cells were harvested (at passage 15), washed using low speed centrifugation (150 × g, 10 minutes) and ressuspended in complete medium. Then, cells were cyto-centrifugated on glass slides, dried at room temperature for 15 minutes and fixed in cold acetone for 15 minutes at -20°C. After a thorough wash with 0.5% BSA in PBS, the cells were treated with polyclonal rabbit anti-GFAP, monoclonal anti-vimentin and polyclonal rabbit anti-S100 antibodies according to the manufactures' instructions. The bound primary antibody was detected using peroxidase labeled polymer conjugated to either mouse or rabbit secondary antibodies. Subsequently, the slides were incubated with a substrate mixture of 3,3-diaminobenzidine (DAB) and 0.02% H_2_O_2_. Cells were then counterstained with haematoxilin and eosin (HE). Control slides that stain positively for the specific antigens were used to assure correct staining and stability of reagents used. Negative controls included the omission of the primary antibody.

### Phase Contrast Microscopy

Growing cells on cover slips were observed with a phase-contrast microscope (Olympus IX50 with a PMC35Dx photo micrographic system).

### Scanning Electron Microscopy

NG97 cells were grown to sub confluence on 13 mm round cover slip in complete medium. The cells were fixed with 2.5% glutaraldehyde and 4% paraformaldehyde in phosphate buffer (pH 7.4) for 1 hour at room temperature. Then, the cells were post-fixed in 1.0% osmium tetroxide (OsO_4_) for 10 minutes, washed in 0.1 M phosphate buffer (pH 7.2) and dehydrated in a grade series of ethanol. Cover slips were critically point dried using liquid CO_2 _as transition fluid. The specimens were cold sputter coated with gold and observed in a JEOL JMS 5800 LV scanning electron microscope (SEM) accelerating voltage of 10 kV.

### Growth Curve

NG97 cells were collected from 13^th ^passage for determination of growth curves. Briefly, semi confluent cultures were trypsinized and cells resuspended in complete medium for counting. Cells (1 × 10^4^) were plated into each well of a 12-well plate and counts from triplicate wells were made daily for 10 days. Trypsinized cells were counted in hemacytometer chamber and numbers were averaged for each time interval. Cell population doubling time was calculated from the linear phase of the growth curve, and the saturation density was the plateau point on the growth curve after the linear growth phase.

## Competing interests

The author(s) declare that they have no competing interests.

## Authors' contributions

This work is part of a Master's Dissertation by Camila M.L. Machado
